# Mycobacterial Diseases of Animals 2012

**DOI:** 10.1155/2012/684720

**Published:** 2012-11-14

**Authors:** Mitchell V. Palmer, Michael D. Welsh, Jesse M. Hostetter

**Affiliations:** ^1^Bacterial Diseases of Livestock, National Animal Disease Center, USDA, Ames, IA 50010, USA; ^2^Agri-Food and Biosciences Institute, Virology Branch, Veterinary Sciences Division, Belfast BT4 3SD, UK; ^3^Department of Veterinary Pathology, College of Veterinary Medicine, Iowa State University, Ames, IA 50011, USA

*Mycobacterium tuberculosis, Mycobacterium bovis, Mycobacterium avium* subsp. *avium, Mycobacterium avium* subsp. *paratuberculosis, Mycobacterium ulcerans,* and other mycobacteria are the etiologies of important diseases in humans and a wide range of animal species including cattle, sheep, goats, deer, possums, badgers, wild boar, elephants, dogs, cats, birds, amphibians, and fish. Moreover, *M. bovis* has a remarkably broad host range and is a serious zoonotic pathogen. In spite of centuries of investigation and costly eradication efforts, *M. bovis* remains an important pathogen at the interface of humans, livestock, and wildlife. *M. avium* subsp. *paratuberculosis* is one of the most widespread pathogens of dairy cattle, causing decreased production, protracted diarrhea, and emaciation.

This 2nd special issue on mycobacterial diseases of animals contains 16 papers comprising 4 reviews and 12 original research papers on various topics including immunology, epidemiology, microbiology, genomics, vaccinology, and pathology. Authors from 9 different countries provide a diverse examination of mixed topics including *M. tuberculosis*, *M. bovis,* and *M. avium* subsp. *paratuberculosis*.

The issue begins with G. M. Kassa et al. examining a cross-section of Ethiopian small ruminants and describing the isolation of *M. tuberculosis* from goats and stressing important zoonotic implications. From Spain, B. Beltrán-Beck and colleagues review the significant progress on oral vaccination of Eurasian wild boar with the vaccine *M. bovis* BCG and a new heat-killed *M. bovis* vaccine. R. A. Skuce et al. review important risk factors for bovine TB herd breakdowns in the United Kingdom and Ireland. The next 3 papers focus on immunology and diagnostics beginning with B. Fernández et al. and their analysis of an ELISA for bovine paratuberculosis. J. T. Nelson and colleagues evaluate a rapid serologic test for tuberculosis diagnosis in several deer species, while M. L. Mon et al. search for improved antigens for use in bovine paratuberculosis diagnosis.

M. V. Palmer and collaborators review in detail wildlife reservoirs of *M. bovis* and the potential for disease transmission at the livestock-wildlife-human interface. C. Mackintosh and colleagues from New Zealand describe experimental infection of red deer with *M. avium* subsp. *paratuberculosis* and examine the clinical, immunological, and pathological outcomes. Rapid diagnostic tests for both bovine tuberculosis and paratuberculosis are high priorities in the veterinary medical field, and A. Wadhwa et al. review current and future platforms for diagnostic assays. A. Lim and colleagues at Michigan State University use genomics to examine differential gene expression between true *M. bovis*-positive and -false-positive cattle.

*Mycobacterium bovis* can infect numerous species, but not all species have maintenance host potential. Intraspecies transmission is requisite to maintenance host potential. Fenton et al. examine potential intraspecies transmission among Virginia opossums in an experimental aerosol challenge model. Possible species differences in susceptibility to *M. bovis* infection lead G. Ameni et al. to explore potential differences in immune responses to *M. bovis* between *Bos indicus* and *Bos taurus* cattle in Ethiopia. C. Furphy and colleagues from Ireland use the molecular epidemiological tools of spoligotyping and VNTR to examine strain types of *M. bovis* in badgers and show that individual badgers can be infected with multiple strains of *M. bovis*.

One useful serological assay for tuberculosis in cattle is the multiantigen print immunoassay, which S. D. Fitzgerald et al. examine, using various interpretation criteria and suggest its use as a possible supplemental test to intradermal tuberculin testing. K. P. Lyashchenko and colleagues describe the uncommon infection of a horse with *M. tuberculosis* and discuss possible zoonotic concerns. The highlands of Cameroon are the setting for J. Awah-Ndukum and collaborators as they use traditional tuberculin skin testing and novel serological tests to determine the prevalence of bovine tuberculosis and explore possible modifications of test interpretation to improve disease surveillance.

The editors thank the many authors for their efforts in the experimentation, labor, and time reflected in each manuscript. The lead editor thanks all editors for time spent reviewing, assigning reviews, and commenting on the many manuscripts submitted. It is the hope of the editors that this 2012 issue will prove useful to investigators, policy makers, and veterinarians involved in the study of mycobacterial diseases of animals.

*Mitchell V. Palmer* *Mitchell V. Palmer* *Michael D. Welsh* *Michael D. Welsh* *Jesse M. Hostetter* *Jesse M. Hostetter*

